# The WISHED Randomized Controlled Trial: Impact of an Interactive Health Communication Application on Home Dialysis Use in People With Chronic Kidney Disease

**DOI:** 10.1177/20543581211019631

**Published:** 2021-06-04

**Authors:** Amber O. Molnar, Andrea Harvey, Michael Walsh, Arsh K. Jain, Eric Bosch, K. Scott Brimble

**Affiliations:** 1 Division of Nephrology, Department of Medicine, McMaster University, Hamilton, ON, Canada; 2 Department of Health Research Methods, Evidence and Impact, McMaster University, Hamilton, ON, Canada; 3 Population Health Research Institute, Hamilton, ON, Canada; 4 University of Toronto, ON, Canada; 5 Division of Nephrology, Department of Medicine, Western University, London, ON, Canada; 6 Eric Bosch Consulting Inc, Hamilton, ON, Canada

**Keywords:** chronic kidney disease, trial, dialysis, education, home dialysis

## Abstract

**Background:**

While home dialysis therapies are more cost effective and may offer improved health-related quality of life, uptake compared to in-center hemodialysis remains low.

**Objective:**

To test whether a web-based interactive health communication application (IHCA) compared to usual care would increase home dialysis use.

**Design:**

Randomized control trial

**Setting:**

Patients were recruited from 3 multidisciplinary kidney clinics across Ontario, Canada (Hamilton, Kingston, London).

**Patients:**

We included adults with advanced chronic kidney disease (CKD) followed in multidisciplinary kidney clinics. Patients who had not completed dialysis modality education, who did not have access to a home computer or the internet, who had significant hearing or vision impairment, who could not read/write/speak English, who had a medical contraindication for home dialysis, or who had selected conservative kidney care were excluded.

**Measurements:**

The primary outcome was any use of home dialysis (peritoneal dialysis or home hemodialysis) within 90 days of dialysis initiation. Secondary outcomes were social support, decision conflict and dialysis knowledge measured at baseline, 6 months and 1 year.

**Methods:**

Eligible patients were randomized to either usual care or the IHCA in addition to usual care in a 1:1 ratio. As part of usual care, all patients received education about dialysis modalities and kidney transplantation delivered by clinic nurses according to local practices. Randomization was performed using a computer-generated sequence in randomly permuted block sizes, stratified by site, and allocation occurred using sequentially numbered sealed, opaque envelopes. Participants, care providers, and outcome assessors were not blinded to the intervention. All analyses were performed blinded using an intention to treat approach. We estimated the effect of the ICHA on the odds of the primary outcome using unadjusted logistic regression models. Linear mixed models for repeated measures over time were used to analyze the impact of the IHCA on the secondary outcomes of interest.

**Results:**

We randomized 140 (usual care, n = 71; IHCA, n = 69) out of a planned 264 patients (mean [SD] age 61 [14.5] years, 65% men). Among patients randomized to the IHCA group that completed 6-month and 1-year follow-up visits, 56.8% and 71.4%, respectively, had not accessed the IHCA website within the past month. There were 23 (32.4%) and 26 (37.7%) patients in the usual care and IHCA groups who received a home dialysis therapy within 90 days of dialysis initiation (odds ratio, OR = 1.3, 95% CI = [0.6-2.5], *P* = .5). Among the 78 patients who initiated dialysis (n = 38 usual care, n = 40 IHCA), 60.5% and 65% in the usual care and IHCA groups received a home therapy within 90 days of dialysis initiation (OR = 1.2, 95% CI = [0.5-3.0], *P* = .7). Secondary outcomes did not differ by intervention group over time.

**Limitations:**

The trial was underpowered due to poor recruitment and use of the IHCA was low.

**Conclusions:**

We did not find evidence of a difference in home dialysis uptake with IHCA use, but our analyses were notably underpowered. The incorporation of greater patient engagement, qualitative research and design research, and pilot implementation may help future evaluations of strategies to improve home dialysis uptake.

**Trial Registration:**

**ClinicalTrials.gov #**NCT01403454, registration date: Jul 21, 2011

## Abrégé

**Contexte:**

La dialyse à domicile est une modalité plus économique qui améliore la qualité de vie liée à la santé des patients. Malgré cela, son adoption demeure faible par rapport à l’hémodialyse en centre hospitalier.

**Objectif:**

Vérifier si l’utilization d’une application interactive de communication en santé (AICS) accessible en ligne augmente l’adoption de la dialyse à domicile comparativement aux soins habituels.

**Type d’étude:**

Essai contrôlé à répartition aléatoire.

**Cadre:**

Les patients ont été recrutés dans trois cliniques multidisciplinaires de néphrologie en Ontario, au Canada (Hamilton, Kingston, London).

**Sujets:**

Nous avons inclus des adultes atteints d’insuffisance rénale chronique (IRC) de stade avancé suivis en clinique multidisciplinaire de néphrologie. Ont été exclus les patients n’ayant pas terminé la formation sur les modalités de dialyse, n’ayant pas accès à un ordinateur ou à Internet à domicile, ayant une déficience auditive ou visuelle importante, ne sachant pas lire, écrire ou parler en anglais, présentant une contre-indication médicale à la dialyse à domicile ou ayant choisi un traitement conservateur.

**Mesures:**

Le principal résultat était utilisation de la dialyse à domicile (dialyse péritonéale ou hémodialyse) dans les 90 jours suivant l’amorce du traitement. Les critères d’évaluation secondaires étaient le soutien social, les conflits décisionnels et les connaissances en matière de dialyse mesurés à l’inclusion, après six mois et après un an.

**Méthodologie:**

Les patients admissibles ont été répartis aléatoirement dans un rapport 1:1 pour recevoir les soins habituels ou l’AICS en plus des soins habituels. Dans le cadre des soins habituels, tous les patients ont reçu une formation sur les modalités de dialyse et la transplantation rénale donnée par le personnel infirmier de la clinique, conformément aux pratiques locales. La répartition aléatoire a été effectuée à l’aide d’une séquence générée par ordinateur dans des blocs de taille permutée au hasard, stratifiés par site. L’attribution s’est faite par enveloppes scellées et opaques, numérotées de façon séquentielle. L’intervention ne s’est pas faite à l’insu des participants, des fournisseurs de soins et des évaluateurs. Toutes les analyses ont été effectuées en aveugle avec une approche en intention de traiter. Nous avons estimé l’effet de l’AICS sur les probabilités du résultat principal en utilisant des modèles de régression logistique non corrigés. Des modèles mixtes linéaires pour des mesures répétées dans le temps ont été employés pour analyser l’impact de l’AICS sur les critères secondaires d’intérêt.

**Résultats:**

Des 264 patients prévus au départ (âge moyen [ÉT]: 61 [14,5] ans; 65 % d’hommes), 140 ont été répartis aléatoirement (71 en soins habituels; 69 avec l’AICS). Dans le groupe AICS, 56,8 % des patients ayant eu une visite de suivi après six mois et 71,4 % après un an n’avaient pas accédé au site Web de l’AICS au cours du mois précédent. Le nombre de patients ayant adopté la dialyse à domicile dans les 90 jours s’élevait à 23 (32,4 %) dans le groupe des soins habituels et à 26 (37,7 %) dans le groupe AICS (risque relatif [RR]: 1,3; IC 95 %: 0,6-2,5; p=0,5). Des 78 patients ayant amorcé des traitements de dialyse (38 en soins habituels; 40 avec l’AICS), la proportion de patients ayant reçu la dialyse à domicile dans les 90 suivants s’élevait à 60,5 % dans le groupe des soins habituels et à 65 % dans le groupe AICS (RR: 1,2; IC 95 %: 0,5-3,0; p=0,7). Les critères d’évaluation secondaires n’ont pas différé au fil du temps selon le groupe d’intervention.

**Limites:**

L’essai manque de robustesse en raison du faible recrutement et de la faible utilisation de l’AICS.

**Conclusion:**

Nous n’avons trouvé aucune preuve supportant une plus grande adoption de la dialyse à domicile avec l’AICS, mais nos analyses manquaient nettement de robustesse. Éventuellement, une plus grande participation des patients, une recherche qualificative et une recherche conceptuelle et une mise en œuvre pilote pourraient faciliter l’évaluation des stratégies visant à augmenter l’adoption de la dialyse à domicile.

## Introduction

Home dialysis (peritoneal dialysis or home hemodialysis) is associated with improvement in certain health-related quality-of-life domains and is cost-effective compared to in-center hemodialysis.^1-4^ Patients and caregivers associate home dialysis with improved freedom, flexibility, and well-being,^5,6^ and a recent survey showed nephrologists believe that home dialysis is better for patients.^7^ Despite this, only a minority of Canadian patients with kidney failure receive home dialysis; less than 20% of new dialysis patients initiate peritoneal dialysis and less than 1% initiate home hemodialysis.^8^ A number of barriers to home dialysis use have been identified, including: patient lack of self-efficacy in performing the therapy, burden on family members, and fear of a catastrophic event.^9^ Absolute medical contraindications to home dialysis are uncommon.^7^ Patient education is clearly recognized as a critical component of dialysis modality selection, and all kidney programs across Canada provide some form of modality education.^10^ Observational studies show that structured pre-dialysis education is associated with high home dialysis uptake (40%-56%), but this has not been tested in randomized controlled trials (RCT).^11,12^ A small RCT (n = 70) showed a 2-phase educational program in patients with CKD increased the intention to initiate home dialysis. Unfortunately, follow-up for actual initiated dialysis modality was not performed,^13^ and evidence suggests a disconnect between intended and initiated dialysis modality.^14,15^ Interactive health communication applications (IHCA) are computer-based information packages for patients that combine health information with at least one of the following: social support, decision support, or behavioral change support. IHCAs have had a positive impact on various outcomes in other chronic diseases, such as diabetes.^16^ Given this, we conducted an RCT to test a web-based IHCA to increase home dialysis uptake among patients with advanced CKD followed in multidisciplinary kidney clinics.

## Methods

### Study Design and Randomization

We conducted a multi-center, parallel group, RCT comparing the use of a secure, web-based IHCA designed specifically for this study versus usual care in the promotion of home dialysis therapies (protocol previously published).^17^ Approval to conduct the trial was obtained by each local institutional Research Ethics Board. The WISHED trial was registered at the National Institutes of Health (ClinicalTrials.gov) #NCT01403454, registration date July 21, 2011. The conduct, design, and reporting of this trial follows the CONSORT statement and recommendations (checklist in Supplemental Appendix A).^18^

Randomization was performed using a computer-generated sequence in randomly permuted block sizes, stratified by site, and allocation occurred using sequentially numbered sealed, opaque envelopes. Only the blinded statistician who prepared the randomization lists knew the randomization sequence and block size. Participants, care providers, and outcome assessors were not blinded to the intervention.

### Setting

The intervention was administered in 3 multidisciplinary kidney clinics in Hamilton, Kingston and London, Ontario, Canada. Each kidney clinic is an academic regional referral center for patients requiring outpatient kidney care, including the management of CKD, dialysis, and kidney transplantation. At the time of the trial, there were approximately 2,300 patients registered in multidisciplinary kidney clinics across the 3 centers.

### Participants

Adult patients (≥18 years) who had received dialysis modality education, had access to a home computer with internet, had declared an intention for dialysis or kidney transplantation (rather than conservative kidney care), and who provided informed consent were potentially eligible. Patients with an absolute medical contraindication to home dialysis, unable to use a home computer or the internet, unable to read, write or speak English, or who had severe visual or auditory impairment were excluded. Eligible patients were randomized to either usual care or the IHCA in a 1:1 ratio. Participants were recruited from February 2012 to September 2016.

The following data were collected at baseline: demographics, living situation, education, comorbidities, blood pressure, body mass index, cause of kidney disease, kidney function, intended kidney replacement therapy, laboratory parameters, hand grip strength and physician assessment as measures of frailty,^19^ Montreal Cognitive Assessment (MoCA) score,^20^ and health literacy measured using the Rapid Assessment of Adult Literacy in Medicine Short Form (REALM-SF) tool.^21^

### Procedures

All participants received usual care in the multidisciplinary kidney clinic. Nephrologists, pharmacists, nurses, social workers, and dietitians delivered usual care. This care included education about dialysis modalities and kidney transplantation delivered by clinic nurses according to local practices.

Participants in the IHCA group were provided an orientation session for the website at the randomization visit and were asked to log on to the website at least monthly in addition to receiving usual care provided in the multidisciplinary kidney clinic. Email reminders to use the website were sent monthly and the frequency of participants’ visits was monitored. The content of the website was developed by nephrologists, dialysis nurses, a nurse educator, a social worker, a dietitian, a nurse manager, dialysis technicians, and home dialysis patients and implemented by a web designer to ensure easy navigation for participants. The goal of the IHCA was to provide educational content about dialysis (with an emphasis on home dialysis) and social support with the goal of reducing decisional conflict and enhancing shared decision-making. The website included *Frequently Asked Questions*, demonstration videos, and still photographs of dialysis equipment, as well as pre-recorded video interviews with local home dialysis experts and existing home dialysis patients. To encourage participant engagement, healthcare professionals added information regularly.

The social support component of the website included video and text narratives of patients discussing their experiences with home dialysis, including perceived benefits and challenges, and a moderated forum for participants to discuss questions about home dialysis with current home dialysis patients. Participants also had the opportunity to email questions to content experts, including nephrologists, nurses, and existing home dialysis patients. The resources were available to all participants randomized to the intervention group, regardless of their intended modality (further details regarding the IHCA provided in Supplemental Appendix B).

### Outcomes

Participants were assessed at 6 months and 1 year after randomization and/or at the time of kidney replacement therapy initiation (dialysis or kidney transplant). The original planned maximum follow-up of 1 year was extended due to slow recruitment. Follow-up of each participant occurred until 90 days after dialysis initiation, kidney transplant, death, study end (November 6, 2018), or loss to follow-up. The primary outcome was any dialysis using a home therapy (peritoneal dialysis or home hemodialysis) within 90 days of dialysis initiation. Participants who did not start dialysis, received a pre-emptive kidney transplant, died, withdrew consent, or were lost to follow-up were considered as non-home dialysis outcomes. The primary outcome was examined in a secondary analysis limited to participants who initiated dialysis during the follow-up (n = 78). Secondary outcomes included dialysis knowledge measured using a locally developed assessment tool (available in Supplemental Appendix C), decision conflict measured using the Decision Conflict scale (16 statements each with 5 response categories scored as 0, 25, 50, 75, 100; average score across statements calculated; higher scores indicative of higher decision conflict),^22^ and level of social support measured with the Duke-UNC Functional Social Support questionnaire (8 questions scored on a 1 to 5 scale; average score calculated; higher scores indicative of greater perceived social support).^23^ Questionnaires were administered at baseline, 6 months, and 1 year to participants who had not started dialysis or received a kidney transplant.

### Sample Size

Based on local data, the baseline proportion of patients who use a home dialysis therapy within 90 days among those who initiate dialysis was estimated at 28%. Assuming a 2-sided alpha of 0.05, and 80% power to detect a 22% absolute risk difference in the proportion of participants starting home dialysis between the IHCA and usual care groups, it was estimated that 152 participants would need to initiate dialysis to detect a significant difference between groups. Based on local transition rates to dialysis, we planned to enroll 264 participants over 1 year. A 22% risk difference was selected based on a home dialysis uptake of approximately 50% seen in prior observational studies examining dialysis education interventions and surveys demonstrating that nephrologists consider 50% to be the optimal target for home dialysis proportion.^11,12,24-26^ Due to the nature of the study population and the CKD care model, we anticipated that there would be no loss to follow-up. Unfortunately, the trial had to be halted after 55 months of recruitment and 78 dialysis events due to feasibility concerns. There were ongoing issues with recruitment at all 3 sites, along with very limited use of the web-based IHCA in the intervention group.

### Statistical Analysis

We used SAS statistical software, version 9.4 (SAS Institute Inc, Cary, North Carolina) for data analysis. All analyses were performed in a blinded fashion using an intention to treat approach. The primary outcome of any home dialysis therapy within 90 days of dialysis initiation was examined by estimating the odds ratio (OR) with 95% confidence interval using logistic regression (SAS Proc Logistic), treating usual care as the referent group, and by estimating the absolute risk difference between treatment groups. Linear mixed models for repeated measures over time (SAS Proc Mixed) were used to analyze the impact of the IHCA compared to usual care on the secondary outcomes of dialysis knowledge, social support and decision conflict scores with fixed effects of time, intervention group and the interactions between time and group. The procedure Proc Mixed prevents list-wise deletion due to missing data; therefore, patients with missing values were not excluded from the analysis and all available data were included. All outcomes were examined in secondary analyses adjusting for covariates known or assumed to be (based on investigator opinion) associated with modality choice, [age, sex, diabetes (y/n), heart failure (y/n), income <CAD55,000 per year (y/n), lives alone (y/n), MoCA score, and hand grip strength]. Covariates for adjustment were selected post-hoc. A 2-sided *P* value <.05 was considered significant without adjustment for multiple comparisons.

## Results

**Figure 1.**
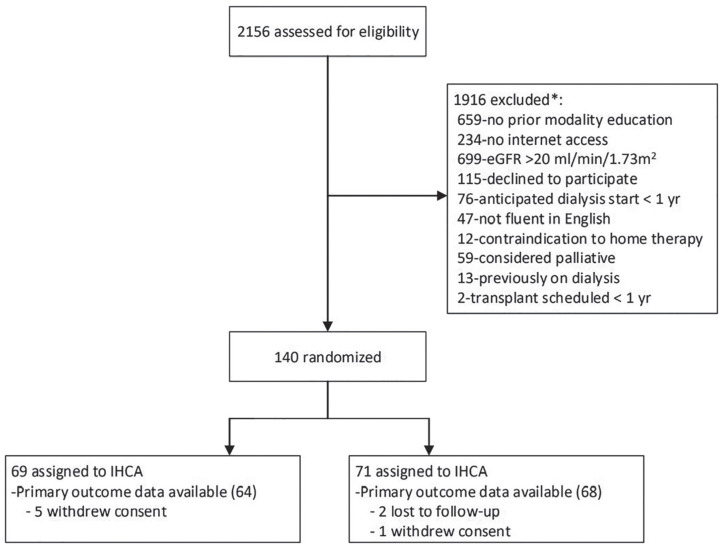
Patient flow diagram. *Note.* IHCA = interactive health communication application. ^*^Screening data only available up to the point that 94 (67.1%) participants were enrolled.

There were 140 participants enrolled, with 71 randomized to the usual care group and 69 to the IHCA group. There were 2 (1.4%) participants lost to follow-up and 6 (4.3%) withdrew consent (Figure 1).

### Baseline Characteristics

The mean (standard deviation, SD) age of all participants was 61 (14.5) years, most were Caucasian (87%), and most were men (65%). The most common cause of CKD was diabetes (41%) and the mean (SD) eGFR was 21 (7.5) mL/min/1.73 m^2^ (Table 1).

**Table 1.** Baseline Characteristics.

Characteristic	Usual care (n = 71)	Interactive health communication application (n = 69)
Age, mean (SD)	59 (14.7)	63 (14.2)
Sex (female), n (%)	28 (39.4)	21 (30.4)
Ethnicity, n (%)		
Caucasian	58 (81.7)	64 (92.8)
Indigenous Peoples	3 (4.2)	1 (1.5)
Hispanic	1 (1.4)	1 (1.5)
Indo-Asian	7 (9.9)	0 (0.0)
Other	2 (2.8)	3 (4.4)
Residence, n (%)		
Apartment	11 (15.5)	8 (11.6)
House	59 (83.1)	56 (81.2)
Other	1 (1.4)	5 (7.3)
Lives alone	8 (11.3)	5 (7.3)
Annual income below CAD55,000, n (%)	42 (59.2)	31 (44.9)
Education level, n (%)^a^		
Did not complete high school	6 (8.5)	10 (14.5)
High school	20 (28.2)	21 (30.4)
College diploma	25 (35.2)	21 (30.4)
University degree	7 (9.9)	7 (10.1)
Graduate or professional degree	13 (18.3)	9 (13.0)
Cause of chronic kidney disease, n (%)		
Diabetes	29 (40.9)	29 (42.0)
Glomerulonephritis	6 (8.5)	7 (10.1)
Polycystic kidney disease	8 (11.3)	6 (8.7)
Vascular	9 (12.7)	13 (18.8)
Other	19 (26.8)	14 (20.3)
Body mass index (kg/m^2^), mean (SD)	30.2 (7.2)	33.2 (13.5)
Hand grip strength (kg), mean (SD)	29 (15.1)	28.6 (13.5)
Frailty based on physician assessment, n (%)^a^		
Very fit	9 (12.7)	5 (7.4)
Well	14 (19.7)	12 (17.7)
Well with treated disease	27 (38.0)	25 (36.8)
Apparently vulnerable	14 (19.7)	19 (27.9)
Mildly frail	4 (5.6)	3 (4.4)
Moderately frail	3 (4.2)	4 (5.9)
Use of a mobility aid, n (%)	12 (16.9)	14 (20.3)
Montreal Cognitive Assessment score, mean (SD)^a,b^	24 (3.8)	24 (3.1)
Diabetes, n (%)	36 (50.7)	33 (47.8)
Hypertension, n (%)	65 (91.6)	67 (97.1)
Systolic blood pressure, mean (SD)	139 (19.7)	137 (16.6)
Diastolic blood pressure, mean (SD)	73 (13.2)	70 (14.2)
Coronary artery disease, n (%)	14 (19.7)	15 (21.7)
Peripheral vascular disease, n (%)	4 (5.6)	5 (7.3)
Amputation, n (%)	2 (2.8)	2 (2.9)
Malignancy, n (%)	7 (9.9)	11 (15.9)
Stroke or transient ischemic attack, n (%)	5 (7.0)	7 (10.1)
Neuropathy, n (%)	8 (11.3)	5 (7.3)
Heart failure, n (%)	6 (8.5)	12 (17.4)
Chronic obstructive pulmonary disease, n (%)	0 (0.0)	6 (8.7)
Arthritis, n (%)	7 (9.9)	6 (8.7)
Serum creatinine, (μmol/L), mean (SD)	295 (83.0)	291 (70.2)
Estimated glomerular filtration rate (mL/min/1.73 m^2^), mean (SD)	21 (7.8)	21 (7.2)
Serum albumin (g/L), mean (SD)	39 (4.8)^c^	41 (3.3)^d^
Rapid Estimate of Adult Literacy in Medicine, Short Form score^e^, mean (SD)	6.8 (0.5)	6.7 (1.0)

*Note.* n = number.

a Missing, n = 1.

b Scores range between 0 and 30; ≥26: normal, <26: mild cognitive impairment or dementia.

c Missing, n = 5.

d Missing, n = 6.

e Score ranges from 0 to 7, 0: less than or equal to third-grade literacy, 1-3: fourth- to sixth-grade literacy, 4-6: seventh to eighth grade literacy, 7: greater than eighth grade literacy.

### Intervention Uptake

At baseline, most participants had not used the internet within the past month to obtain information on dialysis or kidney disease (57.7% usual care, 64.7% IHCA, Supplemental Table 1). Internet use for information on kidney disease was found to be similarly low at follow-up visits in both the usual care and IHCA groups (Table 2). Among participants randomized to the IHCA group who completed 6-month and 1-year follow-up visits, 56.8% and 71.4%, respectively, had not accessed the IHCA website within the past month (Table 2). Among the small number of participants in the IHCA group who reported accessing the website within the past month (n = 19 at 6 months and n = 10 at 1 year), most found the website helpful in learning about home dialysis options and supportive in making a decision about dialysis (Supplemental Table 2).

**Table 2.** Participant Internet and IHCA Use for Information on Kidney Disease as Determined at Follow-Up Visits.

	Never n (%)	Less than once per week n (%)	1–3 times per week n (%)	4–6 times per week n (%)	Every day n (%)
“On average, over the last month how often have you accessed the internet on information about kidney disease or dialysis”? (usual care group)					
6 month visit^a^	36 (66.7)	13 (24.1)	3 (5.6)	2 (3.7)	0 (0.0)
One year visit^b^	33 (66.0)	12 (24.0)	2 (4.0)	2 (4.0)	1 (2.0)
“On average, over the last month how often have you accessed the IHCA website”? (IHCA group)					
6 month visit^c^	25 (56.8)	15 (34.1)	4 (9.1)	0 (0.0)	0 (0.0)
One year visit^d^	25 (71.4)	8 (22.9)	2 (5.7)	0 (0.0)	0 (0.0)
“On average, over the last month how often have you accessed other websites on information about kidney disease or dialysis”? (IHCA group)					
6 month visit^c^	31 (70.5)	9 (20.5)	3 (6.8)	1 (2.3)	0 (0.0)
One year visit^e^	27 (79.4)	3 (8.8)	3 (8.8)	1 (2.9)	0 (0.0)

*Note.* IHCA = interactive health communication application.

a n = 54 respondents.

b n = 50 respondents.

c n = 44 respondents.

d n = 35 respondents.

e n = 34 respondents.

### Primary Outcome

Over a median follow-up of 1.3 (Interquartile range 0.8-2.4) years, 78 (55.7%) participants initiated dialysis (n = 38 usual care, n = 40 IHCA), 15 (10.7%) died prior to dialysis or kidney transplant (n = 5 usual care, n = 10 IHCA), and 6 (4.3%) received a kidney transplant (n = 4 usual care, n = 2 IHCA). There were 23 (32.4%) participants in the usual care group and 26 (37.7%) participants in the IHCA group who received any dialysis using a home therapy (peritoneal dialysis or home hemodialysis) within 90 days of dialysis initiation (absolute risk difference 5.3%). We did not find any difference in home dialysis uptake between the usual care and IHCA arms (OR 1.3, 95% CI = 0.6-2.5, *P* = .5). Results were similar in an adjusted logistic regression analysis (OR 1.5, 95% CI = 0.7-3.2, *P* = .3). Among the subgroup of participants who initiated dialysis over the follow-up, 60.5% in the usual care group and 65.0% in the IHCA group received any dialysis using a home therapy within 90 days of dialysis initiation (absolute risk difference 4.5%). There was no difference in home dialysis uptake between the usual care and IHCA arms (OR 1.2, 95% CI = 0.5-3.0, *P* = .7; Table 3).

**Table 3.** Primary Outcome of Home Dialysis Initiation Within 90 Days.

	Number of events, n (%)		OR (95% CI)	Adjusted OR (95% CI)^a^
	Usual care	Interactive health communication application		
All participants	23 (32.4)	26 (37.7)	1.3 (0.6–2.5)	1.5 (0.7–3.2)
Subgroup of participants who initiated dialysis (N = 78)	23 (60.5)	26 (65.0)	1.2 (0.5–3.0)	1.3 (0.4–3.9)

*Note.* OR = odds ratio; CI = confidence interval.

a Adjusted for age, sex, diabetes (y/n), heart failure (y/n), income <CAD55,000/year (y/n), lives alone (y/n), Montreal Cognitive Assessment score, hand grip strength.

### Kidney Replacement Therapy Planning

At baseline, 90 (64.2%) participants, 45 in each treatment group, intended to do a home dialysis therapy (Table 4). During follow-up, there were 19 (26.8%) and 11 (15.9%) participants in the usual care and IHCA groups who changed their modality plan. Most participants stated that personal choice was the primary reason for changing modality (7 in the usual care and 4 in the IHCA group; Supplemental Table 3).

**Table 4.** Kidney Replacement Therapy Planning at Baseline.

	Usual care (n = 71)	Interactive health communication application (n = 69)
Intended modality, n (%)		
Home dialysis	45 (63.4)	45 (65.2)
Automated peritoneal dialysis	27 (38.0)	27 (39.1)
Continuous ambulatory peritoneal dialysis	8 (11.3)	6 (8.7)
Home hemodialysis	10 (14.1)	12 (17.4)
In-center hemodialysis	9 (12.7)	10 (14.5)
Transplant	10 (14.1)	7 (10.1)
Undecided	7 (9.9)	7 (10.1)
Arterio-venous fistula present	3 (4.2)	8 (11.6)
Peritoneal dialysis catheter inserted	2 (2.8)	1 (1.5)

### Secondary Outcomes

The linear mixed model found that no group showed a statistically significant change in social support score (group-time interaction *P* = .31), decision conflict score (group-time interaction *P* = .90) or dialysis knowledge questionnaire score (group-time interaction *P* = .48; Table 5, Supplemental Figures 1–3). Adjusted results for the linear mixed models were similar, demonstrating no significant change by group or by group-time interaction (Supplemental Table 4).

**Table 5.** Secondary Outcomes Examined Using Linear Mixed Models.

Time	Intervention group		Mixed model analysis with interactions	
	Group Usual care	Group IHCA	Effect	*P* value^a^
Social support^b^				
Baseline	4.6 (0.1) (4.5–4.8)	4.5 (0.1) (4.4–4.7)	Time	.6
6 months	4.6 (0.1) (4.4–4.8)	4.6 (0.1) (4.4–4.8)	Group	1.0
12 months	4.6 (0.1) (4.4–4.7)	4.7 (0.1) (4.5–4.9)	Group × time	.3
Decision conflict^c^				
Baseline	62.3 (0.9) (60.7–64.0)	62.4 (0.9) (60.7–64.1)	Time	.1
6 months	60.4 (1.0) (58.5–62.4)	60.2 (1.1) (58.1–62.3)	Group	.9
12 months	60.5 (1.1) (58.4–62.6)	61.2 (1.2) (58.9–63.6)	Group × time	.9
Dialysis knowledge^d^				
Baseline	46.7 (2.0) (42.7–50.7)	45.0 (2.1) (40.9–49.1)	Time	.2
6 months	50.1 (2.2) (45.8–54.4)	46.3 (2.4) (41.8–50.8)	Group	.5
12 months	48.4 (2.3) (43.6–53.0)	48.5 (2.6) (43.2–53.8)	Group × time	.5

*Note.* Values reported as mean (standard error) with 95% confidence interval unless otherwise specified. *Note.* IHCA = interactive health communication application.

a *P* values associated with type 3 tests of fixed effects.

b Analysis performed on 139 participants, 321 observations. Missing data for 8 participants (n = 3 usual care, n = 5 IHCA). Score ranges from 1 to 5. The higher the average score, the higher the perceived social support.

c Analysis performed on 139 participants, 313 observations. Missing data for 14 participants (n=6 usual care, n=8 IHCA). Score ranges from 0 [no decisional conflict] to 100 [extremely high decisional conflict].

d Analysis performed on 140 participants, 326 observations. Missing data for 7 participants (n = 2 usual care, n = 5 IHCA). Scores reported as percentage of correct responses.

## Discussion

This multi-center randomized controlled trial examined whether offering an IHCA, compared to usual care, increased the uptake of home dialysis in 140 adults with advanced CKD followed in multidisciplinary kidney clinics. We found no effect of the IHCA on the primary outcome of home dialysis therapy received within 90 days of dialysis initiation or any secondary outcome compared to usual care. This trial highlights many of the barriers to evaluating novel methods of effectively engaging patients in modality selection, including low use of the internet in this older population often of lower socioeconomic status, difficulty maintaining patient interest in a web-based intervention, difficulty creating and maintaining engaging information, and the difficulty of executing a complex intervention over a number of years. Whether the lack of notable effect was due to the limited sample size and/or the limited use of the IHCA or whether another IHCA would be effective remains uncertain.

The WISHED trial provides us with several valuable lessons. Recruitment was more difficult than anticipated and use of the intervention was much lower than anticipated. The challenges of conducting trials are well recognized.^27-29^ One proposed method to improve recruitment and retention in clinical research is partnership between researchers and patients at the time of protocol development to obtain and incorporate patient perspectives on the research question and methods and/or have patient volunteers assist with study recruitment.^28^ When the WISHED trial was designed, patient–researcher collaboration was not commonly practiced, and although patient feedback on the intervention was obtained, broader engagement of patients in the early stages of the protocol design may have identified more effective strategies for the recruitment of participants and uptake of the IHCA. A pilot trial may have also helped identify issues with feasibility and acceptability. The information gained from a pilot study could have been used to modify the protocol early on to potentially improve recruitment and uptake of the intervention. Alternatively, a pilot study may have led us to conclude that testing an IHCA in patients with advanced CKD is not feasible and further resources should not be dedicated to pursuing a full trial.^30^ Qualitative work in patients with progressive CKD, which was outside the scope of this study, may help to identify barriers and potential negative perceptions around the use of digital media for disease-specific information and work toward solutions.^31^

Prior studies in patients with CKD suggest an interest in obtaining disease-specific information online,^32-35^ and IHCAs, when studied in other chronic conditions such as asthma and diabetes, showed improvements in disease knowledge, social support, self-efficacy, and hemoglobin A1c levels.^16,36^ However, many studies on digital media use in patients with CKD included kidney transplant recipients, who are generally younger, healthier, and different from non-transplant recipients in a number of ways,^37^ and most prior trials examining IHCAs in chronic disease enrolled young patients.^16^ The multidisciplinary kidney clinic patient population is generally older (mean age for this trial 60 years; mean age for Ontario multidisciplinary kidney clinics 70 years). While general population survey data show that older individuals ≥65 years are increasingly using the internet (59%), use drops off significantly in those older than 75 years, and varies depending on income and education level.^31,38^ As part of this study, we asked participants how often over the past month they accessed the internet for information on kidney disease or dialysis and most (>60%) responded “never.” This suggests that internet use in patients with advanced CKD is much lower than other populations, possibly due to demographics and disease burden; low internet use as a source of information could certainly be a significant contributor to the failure of our web-based intervention.

Across Canada, home dialysis prevalence among patients with kidney failure is low compared to in-center hemodialysis (25% vs 75%).^39^ Home dialysis use in trial participants was much higher than the national average (>60% among patients that started dialysis over the trial follow-up). This may be due to a self-selection bias (ie, trial participants were already more likely to select home dialysis than the general CKD population) and the strict inclusion criteria of the trial. This is relevant to informing the design of future trials in this area—a trial design that minimizes self-selection bias and includes a more generalizable population would help target patients more likely to benefit from the intervention if it is efficacious (ie, high baseline home dialysis use in our study participants suggests a ceiling effect in this population).

We found that most participants (64%) intended to perform a home dialysis therapy at the time of trial enrolment. Despite a very similar proportion of patients intending to do a home therapy and actually initiating a home therapy, it is important to note that 21% of patients changed their mind regarding dialysis modality or kidney transplant over the follow-up. Unfortunately, we did not collect data on the details of the direction of modality decision changes. We have previously demonstrated a disconnect between intended and ultimate dialysis modality.^15^ We found that the most common reasons for a change in modality were personal choice and further education, suggesting patient knowledge, lifestyle, and personal values are critical to this decision, which is consistent with prior studies.^40,41^ The finding of low dialysis knowledge (questionnaire scores <50%) and moderate-high decisional conflict scores throughout the trial show the gaps in our current, standard modality educational practices, and how difficult this decision is for most patients.^42^

In conclusion, our primary objective was to evaluate whether offering an IHCA over usual care affected home dialysis uptake in patients with advanced CKD approaching dialysis. The IHCA we implemented did not alter the choice of kidney replacement therapy. Although the study did not recruit its target sample size, it is unlikely a larger trial would have found an effect as the IHCA was poorly used. The incorporation of a greater extent of patient engagement, qualitative research and design research, and pilot implementation may help future evaluations of these complex, innovative patient education and support tools to increase home dialysis.

## Supplemental Material

sj-pdf-1-cjk-10.1177_20543581211019631 – Supplemental material for The WISHED Randomized Controlled Trial: Impact of an Interactive Health Communication Application on Home Dialysis Use in People With Chronic Kidney Disease Click here for additional data file. Supplemental material, sj-pdf-1-cjk-10.1177_20543581211019631 for The WISHED Randomized Controlled Trial: Impact of an Interactive Health Communication Application on Home Dialysis Use in People With Chronic Kidney Disease by Amber O. Molnar, Andrea Harvey, Michael Walsh, Arsh K. Jain, Eric Bosch and K. Scott Brimble in Canadian Journal of Kidney Health and Disease

sj-pdf-2-cjk-10.1177_20543581211019631 – Supplemental material for The WISHED Randomized Controlled Trial: Impact of an Interactive Health Communication Application on Home Dialysis Use in People With Chronic Kidney Disease Click here for additional data file. Supplemental material, sj-pdf-2-cjk-10.1177_20543581211019631 for The WISHED Randomized Controlled Trial: Impact of an Interactive Health Communication Application on Home Dialysis Use in People With Chronic Kidney Disease by Amber O. Molnar, Andrea Harvey, Michael Walsh, Arsh K. Jain, Eric Bosch and K. Scott Brimble in Canadian Journal of Kidney Health and Disease

sj-pdf-3-cjk-10.1177_20543581211019631 – Supplemental material for The WISHED Randomized Controlled Trial: Impact of an Interactive Health Communication Application on Home Dialysis Use in People With Chronic Kidney Disease Click here for additional data file. Supplemental material, sj-pdf-3-cjk-10.1177_20543581211019631 for The WISHED Randomized Controlled Trial: Impact of an Interactive Health Communication Application on Home Dialysis Use in People With Chronic Kidney Disease by Amber O. Molnar, Andrea Harvey, Michael Walsh, Arsh K. Jain, Eric Bosch and K. Scott Brimble in Canadian Journal of Kidney Health and Disease

sj-pdf-4-cjk-10.1177_20543581211019631 – Supplemental material for The WISHED Randomized Controlled Trial: Impact of an Interactive Health Communication Application on Home Dialysis Use in People With Chronic Kidney Disease Click here for additional data file. Supplemental material, sj-pdf-4-cjk-10.1177_20543581211019631 for The WISHED Randomized Controlled Trial: Impact of an Interactive Health Communication Application on Home Dialysis Use in People With Chronic Kidney Disease by Amber O. Molnar, Andrea Harvey, Michael Walsh, Arsh K. Jain, Eric Bosch and K. Scott Brimble in Canadian Journal of Kidney Health and Disease
