# Supplementary material for: The WISHED Randomized Controlled Trial: Impact of an Interactive Health Communication Application on Home Dialysis Use in People With Chronic Kidney Disease
Source: Can J Kidney Health Dis. 2021 Jun 4;8:20543581211019631. doi: 10.1177/20543581211019631 (PMC8182179; doi:10.1177/20543581211019631)

Appendix B. Details regarding the interactive health communication application (IHCA) intervention

Domain	WEBSITE content
Social support	- Peer blogs/posts<br>- Moderated forum (Invited group discussion on different posted topics)
Decision support	- Moderated forum (Invited group discussion on different posted topics)<br>- Coaching (participants could reach out more directly to peer or expert)
Information/Education	- Common Questions & Answers<br>- "Ask an expert"<br>- Video interviews with patients and content experts<br>- Blogs and narratives from experts (nurses, nephrologists, social worker, dietitian)<br>- Links to other online home dialysis resources

Screenshots of the WISHED study website (no longer active)

independentdialysis.ca

The WISHED study, promoting independent dialysis

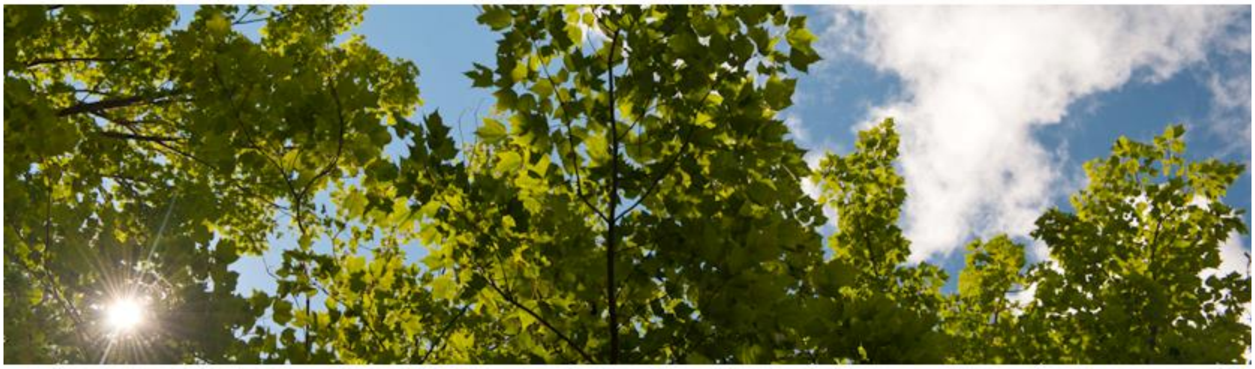

Home

The content of this website is for the exclusive use of randomized participants in the WISHED study being conducted through St. Joseph's Healthcare in Hamilton, Ontario, Canada.

Funding for the WISHED study is provided by the Hamilton Academic Health Sciences Organization (HAHSO) AFP Innovation Fund.

HAHSO
Hamilton Academic Health Sciences Organization

Further access requires a Username and Password. Randomized participants, please use your Username and Password to Log In below.

Username

Password

Maintaining quality

Posted on **November 16, 2016** by **rc**

Making sure that our home PD and HD patients stay safe while at home is a very important goal. Our home program offers a yearly recertification of all skills taught in the initial training. The nurses are able to do this in the home and/or at a clinic appointment. This shows the nurses firsthand the steps our patients use for carrying out dialysis at home (PD or HD). The nurse is able to correct a technique and provide additional information about why it's important to do it a certain way during this re-training session, if required. Quality is important and this is one way our program ensures are patients are staying safe.

-PD nurse

Posted in **Patient safety and precautions** | **Leave a reply** Edit

Fitting dialysis into your life

Posted on **September 27, 2016** by **rc**

Peritoneal dialysis allows you to live your life and enjoy your hobbies. The pictures you see are of a patient that spends weeks at a time on his boat. He has a machine that he is able to leave on the boat during spring/summer/some fall. It still needs to be temperature controlled, so this patient will need to take it off the boat for the winter. He brought his boat from Keswick, Ontario to Grimsby, Ontario in 3 weeks. He is now talking about taking it down the east coast to maybe South Carolina.

You fit dialysis into your life, not your life into dialysis!

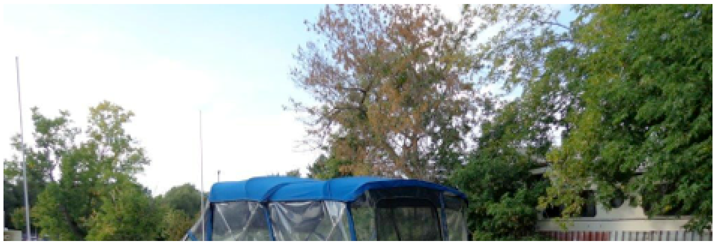

- April 2015
- March 2015
- February 2015
- January 2015
- December 2014
- November 2014
- September 2014
- August 2014
- July 2014
- June 2014
- May 2014
- April 2014
- March 2014
- February 2014
- January 2014
- September 2013
- August 2013
- July 2013
- May 2013
- April 2013
- March 2013
- February 2013
- January 2013
- December 2012
- November 2012
- October 2012
- September 2012
- August 2012
- July 2012
- June 2012
- May 2012
- April 2012
- February 2012

CATEGORIES

- Administration
- AV fistula creation
- Care of your access
- Choosing between PD and HD
- Community supports
- Diet
- Fear of needles
- Financial issues
- Freedom and flexibility
- General
- Hemodialysis

independentdialysis.ca

The WISHED study, promoting independent dialysis

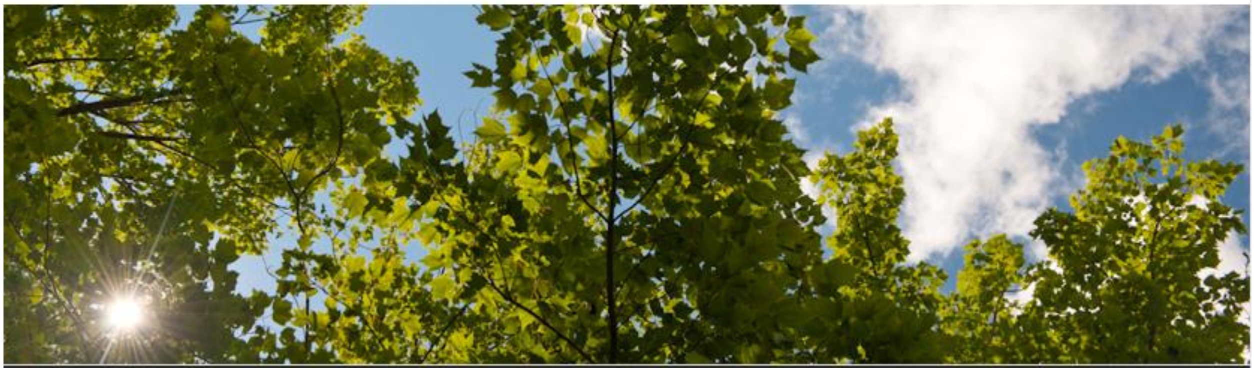

Home Hemodialysis

How does Home Hemodialysis work?

Home Hemodialysis, let's call it Home HD for short, is hemodialysis done at home (obviously!). Let's talk about hemodialysis (HD) first, and then talk about the specifics of Home HD.

HD is a type of dialysis where your blood would be taken from you and cleaned with a machine known as a hemodialysis machine. The machine removes the waste products and excess salt and water from your blood using a hemodialysis cartridge or filter and then returns the cleaned blood to you. This is usually done at the same time three times a week for three to four hours when done in the hospital. When done at home the scheduling can be much more flexible i.e. every day, every other day, or while asleep (nocturnal HD).

One of the main challenges with any form of HD is what is called vascular access. In other words, how do you get the blood from the patient to the machine? The options

Search

RECENT POSTS

- **Website decommissioning**
- **Website maintenance**
- **Maintaining quality**
- **Fitting dialysis into your life**
- **Reliable Renal Diet Resources**

ARCHIVES

- **July 2017**
- **March 2017**
- **November 2016**
- **September 2016**
- **August 2016**
- **June 2016**
- **April 2016**
- **March 2016**
- **February 2016**
- **January 2016**

If you and your doctor decide this is the best type of dialysis for you, you will undergo a same-day procedure where a doctor (usually a surgeon or nephrologist) will place a catheter (see the picture below) into your abdominal space as shown in the picture above, the outside portion of the catheter is tunnelled under your skin and then comes out from the skin so that it can be hooked up as needed. Once the catheter is placed it will be left to heal for a period of time before being used. You will then undergo a period of training to ensure you are ready to do this once you actually start.

Once the catheter is ready to be used it will be connected to a bag of specially made fluid – the fluid then flows into the space in your abdomen and is allowed to simply sit there where it can come in contact with your peritoneal membrane. The fluid is designed to promote the removal of unwanted waste products and excess salt and water over time. After several hours once again you can be connected to a bag to remove the used fluid and a new bag of fluid placed in your abdomen again. Exactly how you do this and how often depend on the type of PD you choose which is discussed in more detail below.

Types of PD

There are 2 main types of PD – CAPD and APD (also called the cycler). These are discussed in more detail below.

CAPD

CAPD stands for continuous ambulatory peritoneal dialysis. CAPD is a manual way of doing PD where, usually four times a day, you carry out an exchange of fluid, removing the old, used fluid and putting the new fluid in. For example, once you are up in the morning you sit down and connect an empty bag to your catheter and drain the used fluid out of your abdomen into the empty bag using gravity. This is commonly referred to as the drain. You then fill your abdomen, again using gravity, with a new bag of fluid that has been connected to your catheter. In actual fact, the empty bag and fresh bag are connected together so that you can do this with one connection rather than two. This is shown in the picture below. This step is known as the fill. Once you have completed the fill you can disconnect, get up and go about your usual daily routine until the next drain and fill. The time that you go about with the fluid in your abdomen is known as the dwell. Most patients will do this three times during the day and a fourth and final time before bed. They then start over the next day and usually do this seven days a week, as the kidney would normally do.

- March 2017
- November 2016
- September 2016
- August 2016
- June 2016
- April 2016
- March 2016
- February 2016
- January 2016
- December 2015
- November 2015
- October 2015
- September 2015
- August 2015
- July 2015
- June 2015
- May 2015
- April 2015
- March 2015
- February 2015
- January 2015
- December 2014
- November 2014
- September 2014
- August 2014
- July 2014
- June 2014
- May 2014
- April 2014
- March 2014
- February 2014
- January 2014
- September 2013
- August 2013
- July 2013
- May 2013
- April 2013
- March 2013
- February 2013
- January 2013
- December 2012
- November 2012
- October 2012
- September 2012
- August 2012
- July 2012
- June 2012
- May 2012
- April 2012

Links

There are many additional sources of information available on the internet. Some of those are listed below. Be sure to also visit our Community Resources for the Patient with Kidney Disease page for information and links specific to services in the Greater Hamilton Area and Ontario.

- Kidney Foundation of Canada | The major Canadian health organization that takes a leadership role in advocating for policies and programs to meet the needs of people living with chronic renal insufficiency.
- Ontario Renal Network | The Ontario Renal Network (ORN) leads a province-wide effort to better organize and manage the delivery of renal services in Ontario.
- American Association of Kidney Patients | The American Association of Kidney Patients (AAKP) is a national non-profit organization founded by kidney patients for kidney patients.
- National Kidney Foundation | The NKF is the major voluntary health organization in the U.S. dedicated to preventing kidney disease, improving the health and well-being of individuals and families affected by kidney disease.
- National Kidney and Urologic Diseases Information Clearinghouse, Peritoneal Dialysis and Home Hemodialysis | A service provided by the US National Institute of Diabetes and Digestive and Kidney Disease at the National Institute of Health.
- British Columbia Renal Agency | The BC Provincial Renal Agency (BCPRA) plans and coordinates health care services for patients with kidney disease in British Columbia. Their goal is to improve the quality of life for those living with kidney disease, while making the best use of health care resources.
- Kidney Community Kitchen | Information and tools to help you manage your renal diet.
- Global Dialysis | Empowering dialysis users and carers.

Search

RECENT POSTS

- **Website decommissioning**
- **Website maintenance**
- **Maintaining quality**
- **Fitting dialysis into your life**
- **Reliable Renal Diet Resources**

ARCHIVES

- **July 2017**
- **March 2017**
- **November 2016**
- **September 2016**
- **August 2016**
- **June 2016**
- **April 2016**
- **March 2016**
- **February 2016**
- **January 2016**
- **December 2015**
- **November 2015**
- **October 2015**
- **September 2015**
- **August 2015**
- **July 2015**
- **June 2015**
- **May 2015**
- **April 2015**
- **March 2015**
- **February 2015**
- **January 2015**
- **December 2014**
- **November 2014**
- **September 2014**
- **August 2014**

Dietary Nutrient Restriction

Dietary Nutrient Restriction/Limit	Standard Hemodialysis (on-site treatment 3x/week)	Peritoneal Dialysis (PD)	Home Hemodialysis (Home HD)	Comparison/ Benefit
**Protein**: 1.2g/kg/day x Ideal Body Weight	High protein intake based on Ideal Body Weight (1.2g/kg/day) **Meats & Alternatives**: Increased intake of chicken, meat, pork, fish, eggs	High protein intake 1.2 – 1.3g/kg/day	High protein intake 1.2 – 1.3g/kg/day	Same protein needs
**Potassium**: Limit to 2500mg/day	Avoid/Limit High Potassium Fruits & Vegetables (ie. oranges, bananas, melon, potato, tomato) **Fruits & Vegetables:** – 3 Servings of Low/Medium Potassium Fruits/day & – 3 Servings of Low/Medium Potassium Vegetables/day	Generally more liberal, sometimes no restriction or a need for increased potassium food intake or potassium supplement	Individual though similar to standard Hemodialysis	Potential for more liberal potassium intake with PD
**Phosphorus**: Limit to ~800-1000mg/day	Avoid other high Phosphorus foods: whole grains, beans & legumes, chocolate, nuts & colas PLUS use of phosphate binder medication daily – Dairy: 1 x ½ cup serving/day – Limit cheese intake to 1oz, 3 times/week	Same Phosphorus restriction as Standard Hemodialysis – Limited Phosphorus food intake PLUS use of phosphate binder medication daily	**Nocturnal Dialysis**: allows for a liberal diet in phosphorus, **Short Daily Sessions or Standard Hemodialysis**: same restriction as Standard Hemodialysis	Potential for more liberal phosphorus intake with nocturnal Home HD
**Sodium**: Limit to ~1500-2300mg/day	No Added Salt	No Added Salt	No Added Salt	Same restriction
**Fluid**:	Usually limited to 1 Litre	Usually no need for	1 Litre fluid limit	Potential for

Search

RECENT POSTS

- Website decommissioning
- Website maintenance
- Maintaining quality
- Fitting dialysis into your life
- Reliable Renal Diet Resources

ARCHIVES

- July 2017
- March 2017
- November 2016
- September 2016
- August 2016
- June 2016
- April 2016
- March 2016
- February 2016
- January 2016
- December 2015
- November 2015
- October 2015
- September 2015
- August 2015
- July 2015
- June 2015
- May 2015
- April 2015
- March 2015
- February 2015
- January 2015
- December 2014
- November 2014
- September 2014
- August 2014
- July 2014
- June 2014
- May 2014
- April 2014
- March 2014
- February 2014
- January 2014

Community Resources for the Patient with Kidney Disease

A number of resources outside of the hospital are available to patients. These are grouped below. Your health care providers, including the social worker, can assist you in identifying your needs and where help may be available. You may also wish to visit the Links page for additional, more general information.

Disease related

- Kidney Foundation, Hamilton and District Chapter, 1 800 387-4474 ext. 4969
- Kidney Foundation, Southwestern Ontario Chapter, 1 800 667-3597, 519 850-5362
- Kidney Foundation, Kingston Chapter, 613 542-2121
- Peer Support (Kidney Foundation), 1 877 202-8222
- Canadian Institute for the Blind, 1 800 563-2642
- Monitoring for Health Program (Diabetes), 1 800 361-0796
- Assistive Devices Program, 1 800 268-6021

Financial

- Ontario Works
  - Hamilton 905 546-4800
  - Brantford 519 759-7009
  - Norfolk 519 426-6170
  - Niagara 905 641-9230
  - Burlington 1-866-4HALTON (1-866-442-5866)
  - Kingston 613 546-2695
- ODSP (Ontario Disability Support Program)
  - Hamilton 905 521-7280
  - Brantford 519 756-5790
  - Burlington 905 637-4500
  - Simcoe 519 426-9350
  - St. Catharines 905 688-3022
  - London 519 438-5111, 1 800 265-4197, teletype: 519 663-5276
  - Kingston 613 545-4553 , 1 800 267 0236, TDD/TTY: (613) 545-4532

Search

RECENT POSTS

- Website decommissioning
- Website maintenance
- Maintaining quality
- Fitting dialysis into your life
- Reliable Renal Diet Resources

ARCHIVES

- July 2017
- March 2017
- November 2016
- September 2016
- August 2016
- June 2016
- April 2016
- March 2016
- February 2016
- January 2016
- December 2015
- November 2015
- October 2015
- September 2015
- August 2015
- July 2015
- June 2015
- May 2015
- April 2015
- March 2015
- February 2015
- January 2015
- December 2014
- November 2014
- September 2014
- August 2014
- July 2014
- June 2014
- May 2014
- April 2014
- March 2014
- February 2014

independentdialysis.ca

The WISHED study, promoting independent dialysis

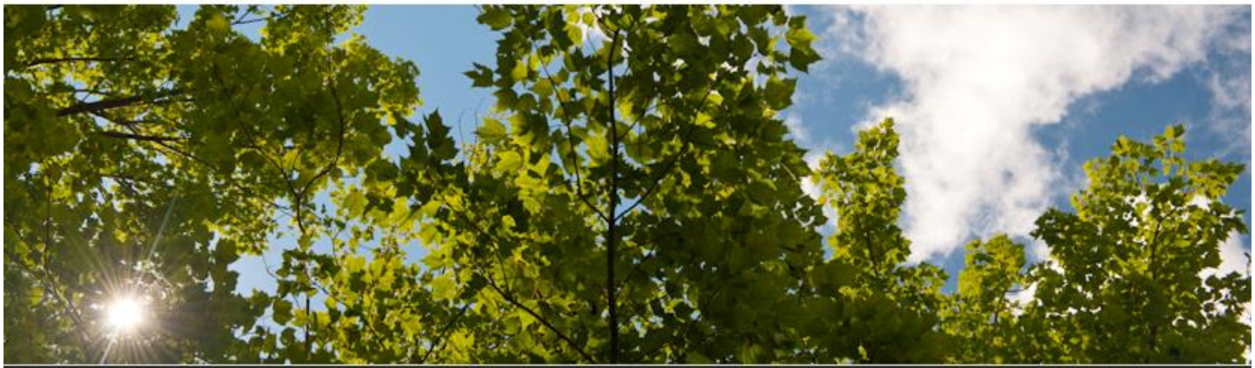

Home Blog Home Hemodialysis Peritoneal Dialysis **Dialysis Resources** Contact Us

Kidney Function Program Doctor

Dr. Rabbat explains the advantages of doing dialysis at home and who is able to do home dialysis. He talks about both peritoneal dialysis and home hemodialysis, and ways to sort out which one might be better for you.

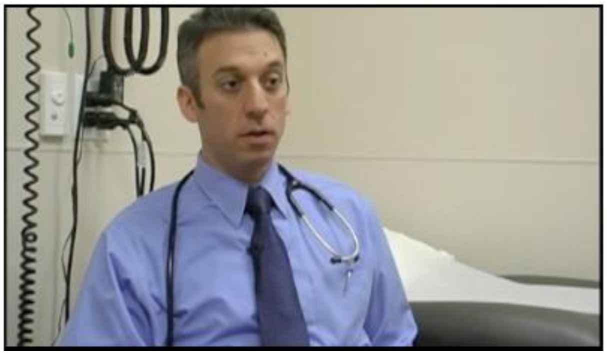

Search

RECENT POSTS

- Website decommissioning
- Website maintenance
- Maintaining quality
- Fitting dialysis into your life
- Reliable Renal Diet Resources

ARCHIVES

- July 2017
- March 2017
- November 2016
- September 2016
- August 2016
- June 2016
- April 2016
- March 2016
- February 2016
- January 2016

Home Blog Home Hemodialysis Peritoneal Dialysis **Dialysis Resources** Contact Us

Interviews

These interviews offer advice for the dialysis patient from the perspective of:

- a Dietitian,
- a Kidney Function Program Doctor and
- a Social Worker.

Search

RECENT POSTS

- Website decommissioning
- Website maintenance
- Maintaining quality
- Fitting dialysis into your life
- Reliable Renal Diet Resources

independentdialysis.ca

The WISHED study, promoting independent dialysis

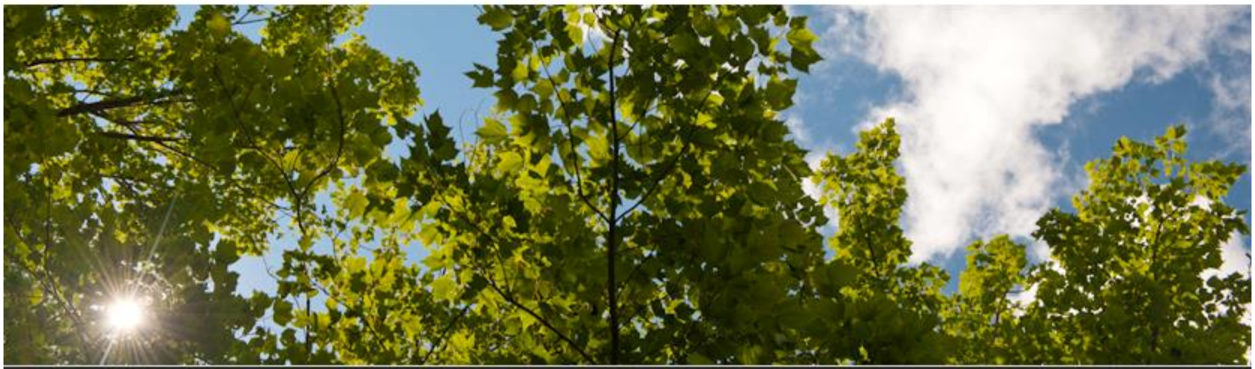

Social Worker

Social worker Gloria Jean Thompson describes what her role is and what types of resources and supports are out there for home dialysis patients. She also discusses whether a home dialysis patient has the ability to continue to work and what supports are available if a patient has to stop working due to illness.

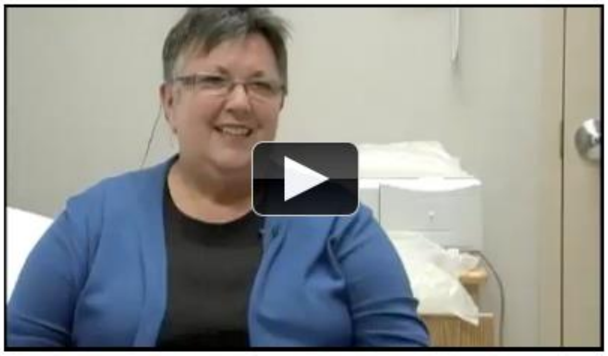

Supplement: sj-pdf-1-cjk-10.1177_20543581211019631 – Supplemental material for The WISHED Randomized Controlled Trial: Impact of an Interactive Health Communication Application on Home Dialysis Use in People With Chronic Kidney Disease [file sj-pdf-1-cjk-10.1177_20543581211019631.pdf]
