# Supplementary material for: The WISHED Randomized Controlled Trial: Impact of an Interactive Health Communication Application on Home Dialysis Use in People With Chronic Kidney Disease
Source: Can J Kidney Health Dis. 2021 Jun 4;8:20543581211019631. doi: 10.1177/20543581211019631 (PMC8182179; doi:10.1177/20543581211019631)
Supplement: sj-pdf-3-cjk-10.1177_20543581211019631 – Supplemental material for The WISHED Randomized Controlled Trial: Impact of an Interactive Health Communication Application on Home Dialysis Use in People With Chronic Kidney Disease [file sj-pdf-3-cjk-10.1177_20543581211019631.pdf]

Appendix C: Dialysis Knowledge Questionnaire

○ Baseline ○ 6-months ○ Study end

Participant ID: ☐☐☐ Date completed (dd/mm/yyyy): ☐☐/☐☐/☐☐☐☐

1) Below are a list of possible symptoms of kidney disease. Please select all of the symptoms a person may have if they have kidney failure?

	**Yes**	**No**
Increased fatigue	☐	☐
Back pain	☐	☐
Metal taste/bad taste in the mouth	☐	☐
Excess itching	☐	☐
Increased appetite	☐	☐

2) How many times a week do most **hemodialysis** patients do their treatments?
   a. 1
   b. 3
   c. 4
   d. 6
   e. None

3) Which of the following is **FALSE** regarding **peritoneal dialysis**?
   a. A peritoneal dialysis tube is placed in the abdomen (belly) to allow for dialysis.
   b. A glucose solution is usually left in the abdomen (belly) for several hours in order for dialysis to occur and then drained
   c. There is a lower risk of low blood pressure with peritoneal dialysis as compared to hemodialysis
   d. Patients performing peritoneal dialysis always require the help of a family member or a nurse as this type of dialysis cannot be done alone
   e. Dialysis is possible because the body uses a lining in the abdomen (belly) to act as a filter called the peritoneum to remove wastes and fluid

4) Below is a list of possible advantages and disadvantages when comparing peritoneal dialysis (PD) to conventional hemodialysis (HD), which is performed in a dialysis unit. Please select for each statement which type of dialysis this is true for?

	**HD**	**PD**
Less dietary and fluid restrictions	☐	☐
Ability to produce urine for a longer time	☐	☐

Appendix C: Dialysis Knowledge Questionnaire

Usually performed 3 times per week	☐	☐
Is easier to travel	☐	☐
Usually requires continual nursing supervision	☐	☐
Allows you to become more independent	☐	☐

5) Possible disadvantages of peritoneal dialysis (PD) include all of the following EXCEPT:
   a. May lead to weight gain
   b. A higher risk of hernias due to higher pressures in the abdomen
   c. Increased risk of an abdominal infection called peritonitis
   d. May increase cholesterol levels
   e. May have decreased quality of life compared to hemodialysis

6) Match the corresponding types of dialysis sessions that can be done at home
   a. Home Standard Hemodialysis __________
   b. Nocturnal Hemodialysis (NHD) __________
   c. Short Daily Hemodialysis (SDHD) __________
   d. Continuous Automated Peritoneal Dialysis (CAPD) __________
   e. Automated Peritoneal Dialysis (APD) __________

   i) Hemodialysis three to four hours 3 times per week
   ii) Two to three hour hemodialysis treatments five to seven days per week
   iii) Four to five fluid exchanges during the day
   iv) Six to nine hour overnight hemodialysis treatments three to seven nights per week
   v) Four to six fluid exchanges overnight using a machine

7) Compared to conventional hemodialysis, nocturnal hemodialysis (NHD) has been found to have additional benefits. Which of the following statements below is **FALSE**?
   a. NHD is associated with improved sleep and improved quality of life
   b. NHD allows for greater flexibility with diet and fluids
   c. NHD patients take less medications since their blood is "cleaned" more efficiently
   d. NHD allows for family planning and provides an opportunity for women to become pregnant and deliver safely
   e. NHD improves kidney function

8) Patients who do home hemodialysis tend to feel better than patients who choose hemodialysis at a dialysis centre.
   a. True
   b. False

9) Which of the following treatments is associated with the best cleaning of your blood
   a. Standard Home Hemodialysis
   b. Nocturnal Hemodialysis (NHD)
   c. Short Daily Hemodialysis (SDHD)
   d. Continuous Automated Peritoneal Dialysis (CAPD)

    e. Automated Peritoneal Dialysis (APD)

10) Which of the following therapies allows you to have the most freedom for traveling and allows you to dialyze **without** a machine?
    a. Standard Home Hemodialysis
    b. Nocturnal Hemodialysis (NHD)
    c. Short Daily Hemodialysis (SDHD)
    d. Continuous Automated Peritoneal Dialysis (CAPD)
    e. Automated Peritoneal Dialysis (APD)

11) All of the following dialysis therapies are recognized as "gentle dialysis" therapies with good control of blood pressure EXCEPT:
    a. Conventional Hemodialysis performed at a dialysis centre
    b. Nocturnal Hemodialysis (NHD)
    c. Short Daily Hemodialysis (SDHD)
    d. Continuous Automated Peritoneal Dialysis (CAPD)
    e. Automated Peritoneal Dialysis (APD)

12) What access can patients use if they do hemodialysis at home?
    a. Arteriovenous Fistula (AVF)
    b. Ateriovenous Graft (AVG)
    c. Central Venous Catheter
    d. All of the above
    e. None of the above

13) Research shows that patients who actively participate in their care have many benefits. Which of the following is **FALSE**?
    a. Better control of symptoms from their illness
    b. A poorer quality of life
    c. Improved energy and less disability
    d. Better communication with their physicians
    e. Less hospitalizations

14) Since there are so many benefits with Home Dialysis, why don't patients choose to dialyze at home? Reasons include all of the following **EXCEPT:**
    a. Poor patient education regarding home-based therapies
    b. Lack of self confidence
    c. Being scared of needles
    d. Patients do not realize all the support they would have
    e. Patients like to be restricted and not be involved in their care

Person completing report: ______________________________

Print last name, first initial **Signature**

Date: [|] / [|] / [| | |]

day / month / yea
