# Supplementary material for: The WISHED Randomized Controlled Trial: Impact of an Interactive Health Communication Application on Home Dialysis Use in People With Chronic Kidney Disease
Source: Can J Kidney Health Dis. 2021 Jun 4;8:20543581211019631. doi: 10.1177/20543581211019631 (PMC8182179; doi:10.1177/20543581211019631)
Supplement: sj-pdf-4-cjk-10.1177_20543581211019631 – Supplemental material for The WISHED Randomized Controlled Trial: Impact of an Interactive Health Communication Application on Home Dialysis Use in People With Chronic Kidney Disease [file sj-pdf-4-cjk-10.1177_20543581211019631.pdf]

Supplemental Table 1: Response to the question: "On average over the last month how often have you accessed the internet on information about dialysis or kidney disease"?

Response at baseline	Usual care<br>n=71	IHCA[a]<br>n=68
Never, n (%)	41 (57.7)	44 (64.7)
Less than once per week, n (%)	18 (25.4)	16 (23.5)
1-3 times per week, n (%)	7 (1.4)	3 (4.4)
4-6 times per week	4 (5.6)	1 (1.5)
Every day, n (%)	1 (1.4)	4 (5.9)

[a]Missing, n=1

Abbreviation: IHCA: interactive health communication application

Supplemental Table 2: Acceptability and usefulness of the IHCA website among participants in the IHCA group who accessed the website within the past month[a]

	Strongly disagree n (%)	Disagree n (%)	Neither agree or disagree n (%)	Agree n (%)	Strongly agree n (%)
I find the IHCA website helpful in learning about home dialysis options					
6 month visit	1 (5.3)	1 (5.3)	2 (10.5)	12 (63.2)	3 (15.8)
One year visit	1 (10.0)	2 (20.0)	0 (0.0)	6 (60.0)	1 (10.0)
I find the IHCA website supportive in helping me make a decision about dialysis					
6 month visit	1 (5.3)	4 (21.1)	2 (10.5)	11 (57.9)	1 (5.3)
One year visit	0 (0.0)	1 (10.0)	1 (10.0)	8 (80.0)	0 (0.0)

[a]n=19 at 6 months and n=10 at 1 year
Abbreviation: IHCA: interactive health communication application

Supplemental Table 3: Reasons for changing modality plan

Reasons	Usual care[a] n (%)	IHCA[b] n (%)
Change in health status, n (%)	1 (5.3)	1 (9.1)
Personal choice, n (%)	7 (36.8)	4 (36.4)
Family influence, n (%)	2 (10.5)	0 (0.0)
Physician, n (%)	0 (0.0)	3 (27.3)
Nursing influence, n (%)	2 (10.5)	1 (9.1)
Change in living situation, n (%)	3 (15.8)	0 (0.0)
Change in health of support person, n (%)	0 (0.0)	0 (0.0)
Further education, n (%)	4 (21.1)	2 (18.2)

[a]19 out of 71 (26.8%) participants randomized to the usual care arm changed their modality plan

[b]11 out of 69 (15.9%) participants randomized to the IHCA arm changed their modality plan

Abbreviation: IHCA: interactive health communication application

Supplemental Table 4: Adjusted linear mixed models[a]

Social Support[b]				
Time	Intervention group		Mixed model analysis with interactions	
	Group IHCA	Group Usual Care	Effect	P value[c]
Baseline	4.4 (0.1) (4.2-4.6)	4.2 (0.1) (4.0-4.4)	Time	0.6
6 months	4.4 (0.1) (4.1-4.6)	4.2 (0.1) (4.0-4.5)	Group	0.4
12 months	4.3 (0.1) (4.1-4.6)	4.4 (0.1) (4.1-4.6)	Group x time	0.3
**Decision Conflict[d]**				
Baseline	62.5 (1.3) (60.0-65.0)	62.4 (1.3) (59.9-65.0)	Time	0.1
6 months	60.6 (1.4) (57.9-63.3)	60.2 (1.4) (57.4-63.0)	Group	1.0
12 months	60.7 (1.5) (57.9-63.6)	61.2 (1.5) (58.2-64.2)	Group x time	0.9
**Dialysis knowledge[e]**				
Baseline	44.6 (2.9) (38.8-50.4)	43.4 (2.9) (37.6-49.2)	Time	0.2
6 months	48.1 (3.1) (42.0-54.2)	44.7 (3.1) (38.6-50.8)	Group	0.6
12 months	46.5 (3.1) (40.3-52.6)	46.9 (3.3) (40.4-53.4)	Group x time	0.5

[a]Values reported as mean (standard error) with 95% confidence interval unless otherwise specified.
[b]Analysis performed on 139 participants, 321 observations. Missing outcome data for 8 participants (n=3 usual care, n=5 IHCA). Score ranges from 1 to 5. The higher the average score, the higher the perceived social support.
[c]P values associated with type 3 tests of fixed effects.
[d]Analysis performed on 139 participants, 313 observations. Missing outcome data for 14 participants (n=6 usual care, n=8 IHCA). Score ranges from 0 [no decisional conflict] to 100 [extremely high decisional conflict].
[e]Analysis performed on 139 participants, 325 observations. Missing outcome data for 7 participants (n=2 usual care, n=5 IHCA). Scores reported as percentage of correct responses.
